# Oxaliplatin Treatment Alters Systemic Immune Responses

**DOI:** 10.1155/2019/4650695

**Published:** 2019-02-18

**Authors:** Vanesa Stojanovska, Monica Prakash, Rachel McQuade, Sarah Fraser, Vasso Apostolopoulos, Samy Sakkal, Kulmira Nurgali

**Affiliations:** ^1^Institute for Health and Sport, Victoria University, Melbourne, VIC 8001, Australia; ^2^Department of Medicine, Western Health, Faculty of Medicine, Dentistry and Health Sciences, The University of Melbourne, Australia

## Abstract

**Purpose:**

Oxaliplatin is a platinum-based chemotherapeutic agent demonstrating significant antitumor efficacy. Unlike conventional anticancer agents which are immunosuppressive, oxaliplatin has the capacity to stimulate immunological effects in response to the presentation of damage associated molecular patterns (DAMPs) elicited upon cell death. However, the effects of oxaliplatin treatment on systemic immune responses remain largely unknown. Aims of this study were to investigate the effects of oxaliplatin treatment on the proportions of (1) splenic T cells, B cells, macrophages, pro-/anti-inflammatory cytokines, gene expression of splenic cytokines, chemokines, and mediators; (2) double-positive and single-positive CD4^+^ and CD8^+^ T thymocytes; (3) bone-marrow hematopoietic stem and progenitor cells.

**Methods:**

Male BALB/c mice received intraperitoneal injections of oxaliplatin (3mg/kg/d) or sterile water tri-weekly for 2 weeks. Leukocyte populations within the spleen, thymus, and bone-marrow were assessed using flow cytometry. RT-PCR was performed to characterise changes in splenic inflammation-associated genes.

**Results:**

Oxaliplatin treatment reduced spleen size and cellularity (CD45^+^ cells), increased the proportion of CD4^+^, CD8^+^, and Treg cells, and elevated TNF-*α* expression. Oxaliplatin was selectively cytotoxic to B cells but had no effect on splenic macrophages. Oxaliplatin treatment altered the gene expression of several cytokines, chemokines, and cell mediators. Oxaliplatin did not deplete double-positive thymocytes but increased the single-positive CD8^+^ subset. There was also an increase in activated (CD69^+^) CD8^+^ T cells. Bone-marrow hematopoietic progenitor pool was demonstrably normal following oxaliplatin treatment when compared to the vehicle-treated cohort.

**Conclusion:**

Oxaliplatin does not cause systemic immunosuppression and, instead, has the capacity to induce beneficial antitumor immune responses.

## 1. Introduction

It is well established that oxaliplatin can evoke the presentation of damage associated molecular patterns (DAMPs) within cancer cells to induce potent immunogenic cell death [1–4]. Despite its immunostimulatory potential, the systemic immune responses following oxaliplatin treatment remain largely unknown. We have previously demonstrated that oxaliplatin treatment causes the nuclear overexpression and cytoplasmic translocation of the DAMP high-mobility group box 1 (HMGB1), within the colon. However, despite the induction of DAMPs, oxaliplatin treatment does not result in gastrointestinal inflammatory responses. We hypothesised that the lack of inflammation within the colon following oxaliplatin treatment is due to tissue-specific responses, rather than immunosuppression by this anticancer agent.

The gastrointestinal mucosa is continuously challenged by a myriad of antigens, pathogens, nutrients, and ions and is a prime target for cytotoxic insult by anticancer agents due to its high proliferation rate [5, 6]. Given the constant exposure to harmful antigens, the gastrointestinal immune system has evolved a level of tolerance against pathogens and antigens [6, 7]. Thus, bouts of inflammation in response to individual stimuli would be detrimental to the host.

The spleen plays a major role in augmenting systemic immune responses to blood borne pathogens and antigens, as it is rich in antigen presenting cells, and effector lymphocytes which produce appropriate adaptive immunological responses [8, 9]. The thymus and bone marrow provide a replenishing pool of leukocytes which migrate to lymphoid organs such as the spleen upon maturation. Currently, there is minimal research documenting the immunological changes within the spleen, thymus, and bone marrow following oxaliplatin treatment; specifically, there is a paucity of studies on the impact of oxaliplatin treatment on haematopoiesis.

The aims of this study were to investigate the effects of oxaliplatin treatment on spleen size and leukocyte cellularity and phenotype. The effects of oxaliplatin treatment in polarising inflammatory cytokine responses were assessed. Thymocytes and bone marrow hematopoietic progenitor and stem cells were studied to determine their role in oxaliplatin-induced changes in leukocytes.

## 2. Materials and Methods

### 2.1. Animals

Male, BALB/c mice (n=47, aged 5-7 weeks, weighing 18-25g) were used in this study. Mice had access to food and water* ad libitum* and were kept under a 12 hour light/dark cycle in a well-ventilated room at a temperature of 22°C. Mice acclimatised for up to 1 week prior to the commencement of* in vivo* intraperitoneal injections. All efforts were made to minimise animal suffering, to reduce the number of animals used and to utilise alternatives to* in vivo* techniques, if available. All procedures in this study were approved by the Victoria University Animal Experimentation Ethics Committee (Ethics No: 15-011) and performed in accordance with the guidelines of the National Health and Medical Research Council Australian Code of Practice for the Care and Use of Animals for Scientific Purposes.

### 2.2. Oxaliplatin Treatment

Mice were separated into 2 cohorts (n=5-15/group): (1) vehicle (sterile water), (2) oxaliplatin (3mg/kg, Sigma-Aldrich, Australia). All mice received intraperitoneal injections (maximum of 200*μ*l/injection) using 26 gauge needles, tri-weekly for up to 14 days. Dosages were calculated per body surface area as per previously published method [10, 11]. We administer oxaliplatin for 2 weeks as this is equivalent to the cumulative chemotherapeutic dose given in humans. We have previously published data describing the neurotoxic effects of oxaliplatin following 2 weeks of treatment [12, 13] and, thus, aimed to determine whether this neurotoxicity is associated with systemic inflammation. Mice were culled via cervical dislocation 14 days subsequent to their first intraperitoneal injection, and spleen, thymus, and bone marrow were harvested.

### 2.3. Flow Cytometry

To identify changes in immune cell composition following oxaliplatin treatment, the spleen, thymus, and bone marrow were harvested. Manual cell suspensions of the spleen and thymus were performed. The bone marrow was harvested using a syringe flush-out method on both hind limbs from each animal. Cell suspensions were centrifuged at 1500 rpm for 5 minutes at 4°C and resuspended in red blood cell lysis buffer (BD Biosciences, USA) and incubated in the dark for 20 minutes. Samples were centrifuged at 1500 rpm for 5 minutes at 4°C. The supernatant of each cell suspension was aspirated and the pellet containing the immune cells was then resuspended in 1 ml of FACS buffer and filtered. Aliquots (10 *μ*l) of each cell suspension were transferred into separate eppendorf tubes containing 10 *μ*l of trypan blue. Manual cell counts were performed using haemocytometer. Cell suspensions with >90% cell viability were only then used for immunolabelling. Cells were transferred appropriately to 96 U-bottom well plates (BD Biosciences, USA) and were centrifuged at 1300 rpm for 3 minutes at 4°C. Subsequent to centrifugation, the 96 U-bottom well plates were then aspirated. A selection of cell surface antibodies was used to identify various immune cell populations (Table 1). For intracellular labelling of cytokines, spleen cell suspensions were permeabilised using a CytoFix/Perm kit (BD Biosciences, USA) according to manufacturer's instructions. Furthermore, 30 *μ*l of each antibody cocktail was loaded to appropriate wells and incubated for 20 minutes at 4°C. Subsequent to the incubation period, cells were washed with 165 *μ*l of FACS buffer and centrifuged at 1300 rpm for 3 minutes at 4°C. The plates were aspirated and cells within each well were resuspended in 200 *μ*l of FACS buffer. Cells were then transferred to FACS tubes. BD Biosciences LSR II or FACS CANTO II flow cytometers were used to collect 200,000 cells from each cell suspension. Data were acquired using BD FACSDiva™ software v6.1 (BD Biosciences, USA), and analysis was conducted using FlowJo (Tree Star, USA) or FACSDiva™ v6.1 software (BD Biosciences, USA). The 7AAD viability marker was not in our flow cytometry antibody panels during acquisition since the viability of our cells were in excess of 90%; thus, we performed exclusion gating to eliminate any dead cells and debris using FSC and SSC gating. These settings have been confirmed to be accurate using 7AAD backgating, a method published previously [14].

### 2.4. RNA Isolation and RT^2^ Profiler PCR

Spleen tissue was removed from vehicle-treated and oxaliplatin-treated mice (n=5/group). Total RNA was extracted using TRIzol™ (Invitrogen, Carlsbad, USA) and further purified using RNeasy® Mini columns (Qiagen, Hilden, Germany), including DNase digestion to remove residual genomic DNA. The integrity of all RNA samples was assessed on an Agilent 2100 Bioanalyzer (Agilent Biotechnologies), setting the limit for inclusion in the study as an RNA Integrity Number (RIN) of 9.0. The concentration of individual RNA samples was measured using a Qubit RNA BR Assay (Invitrogen) and RNA pools were prepared for mRNA expression analysis by combining equal quantities of RNA within each group. Gene expression was investigated with the pathway specific RT^2^ Profiler PCR Array “Mouse Cancer Inflammation and Immunity Crosstalk” (Qiagen, Cat. no. PAMM-181Z), using 0.5 *μ*g pooled RNA template, as described previously [15].

### 2.5. Statistical Analysis

Statistical analysis included the student* t*-test or One-Way ANOVA with multiple comparisons and Bonferroni's post-hoc test using GraphPad Prism™ v6.0 (GraphPad Software, USA). The data were represented as mean ± standard error of the mean (SEM). Statistical significance was defined where the P value was less than 0.05.

## 3. Results

### 3.1. Oxaliplatin Treatment Decreases Spleen Mass

**Figure 1:**
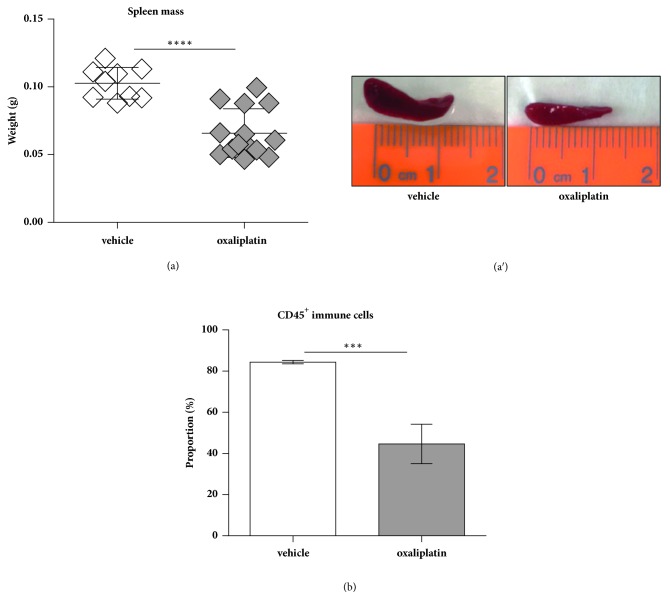
*Effects of oxaliplatin on spleen mass and cellularity*. To investigate toxicity of the spleen following oxaliplatin treatment we measured weight (g). Oxaliplatin treatment caused a significant reduction in spleen mass when compared to those obtained from the vehicle-treated cohort (a, a′). A significant reduction in the proportion of CD45^+^ leukocytes was observed following oxaliplatin treatment when compared to the vehicle-treated group (b). *∗∗∗P*<0.001; *∗∗∗∗P*<0.0001.

To determine toxicity of the spleen following oxaliplatin treatment we measured weight (g). Oxaliplatin treatment caused a significant reduction in spleen mass (0.065g ± 0.004g,* P*<0.0001; n=14) when compared to those obtained from the vehicle-treated cohort (0.102g ± 0.003g; n=9) (Figures 1(a) and 1(a′)). Furthermore, we determined cellularity with respect to CD45^+^ cells using flow cytometry. A significant reduction in the proportion of CD45^+^ leukocytes was noted following oxaliplatin treatment (44.6 ± 4.7%,* P*<0.001; n=4) when compared to the vehicle-treated group (84.4 ± 0.4%; n=4) (Figure 1(b)).

### 3.2. Oxaliplatin Treatment Differentially Affects CD4^+^, CD8^+^, and Treg Populations within the Spleen

**Figure 2:**
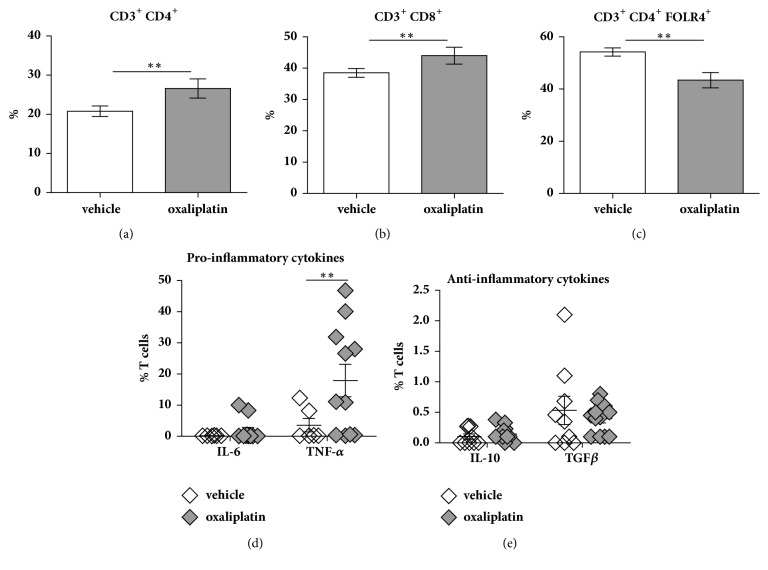
*Effects of oxaliplatin on the proportion of T cell populations and pro-/anti-inflammatory cytokines within the spleen*. To determine any changes in the proportions of CD4^+^ and CD8^+^ T cells and Tregs we gated on CD3^+^CD4^+^/CD8^+^/GR-1^−^/FOLR4^+^ events. Proinflammatory and anti-inflammatory cytokines were gated on IL-6, TNF-*α*, IL-10, and TGF*β* events. Oxaliplatin treatment caused a significant increase in the proportion of CD3^+^ CD4^+^ and CD3^+^ CD8^+^ T cells when compared to the vehicle-treated group (a, b). Conversely, oxaliplatin treatment caused a significant decrease in the proportion of CD3^+^ CD4^+^ FOLR4^+^ T cells when compared to the vehicle-treated group (c). No changes in IL-6 expression were observed between the vehicle-treated and the oxaliplatin-treated mice; however, a significant increase in TNF-*α* was noted in the oxaliplatin-treated group when compared to the vehicle-treated cohort (d). No changes in either anti-inflammatory cytokines were observed between the vehicle-treated and oxaliplatin-treated mice (e). Vehicle: n=5-9; oxaliplatin: n=5-14; *∗∗P*<0.01.

To determine any changes in the proportions of CD4^+^ and CD8^+^ T cells, we gated on CD3^+^CD4^+^/CD8^+^/GR-1^−^/FOLR4^+^ expressing cells and cytokines (pro-inflammatory: IL-6 and TNF-*α*; anti-inflammatory: IL-10 and TGF*β*). Oxaliplatin treatment caused a significant increase in the proportion of CD4^+^ T cells (26.6 ± 1.1%,* P*<0.01; n=5) when compared to the vehicle-treated cohort (20.8 ± 0.6%; n=5) (Figure 2(a)). Oxaliplatin treatment also caused a significant increase in the proportion of CD8^+^ T cells (44.0 ± 0.9%,* P*<0.01; n=9) when compared to the vehicle-treated group (38.5 ± 0.7%; n=4) (Figure 2(b)). Furthermore, a significant reduction in the proportion of regulatory T cells (Tregs) was noted following oxaliplatin treatment (43.4 ± 1.3%,* P*<0.01; n=5) when compared to the vehicle-treated cohort (54.2 ± 0.7%; n=5) (Figure 2(c)).

No changes in IL-6 expression of T cells from the spleen was observed between the vehicle-treated (0.20 ± 0.01%; n=9) and the oxaliplatin-treated mice (1.80 ± 0.97%; n=14) (Figure 2(d)). However, a significant increase in TNF-*α* was observed in the oxaliplatin-treated group (17.9 ± 4.6%;* P*<0.01; n=14) when compared to the vehicle-treated cohort (3.5 ± 1.4%; n=9) (Figure 2(d)). There were no changes to anti-inflammatory cytokines between vehicle-treated (IL-10: 0.10 ± 0.03%; n=9; TGF-*β*: 0.5 ± 0.2%; n=9) and oxaliplatin-treated mice (IL-10: 0.14 ± 0.03%; n=14; TGF-*β*: 0.39 ± 0.06%; n=14) (Figure 2(e)).

**Figure 3:**
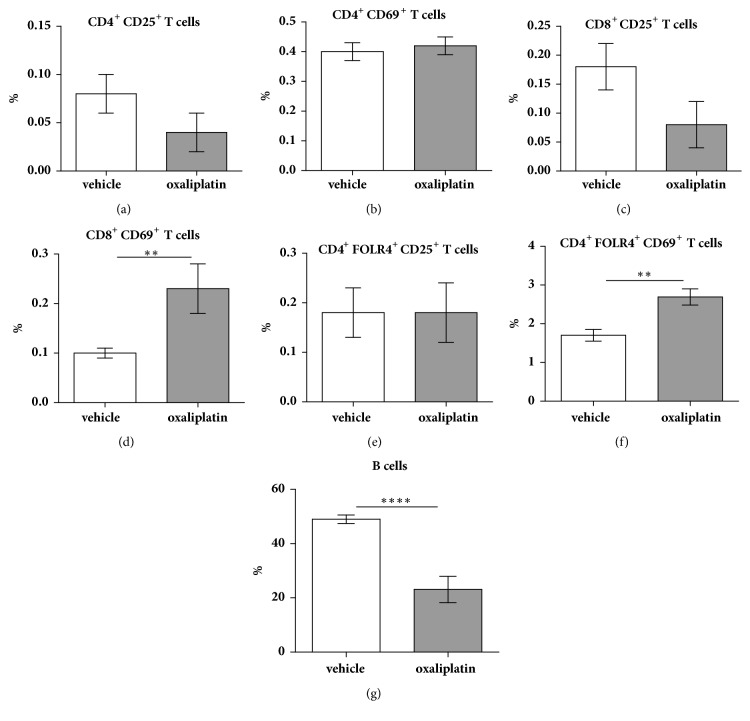
*Effects of oxaliplatin on the proportion of activated T cell populations and B cells within the spleen*. To determine any changes in the proportions of activated CD4^+^ and CD8^+^ T cells, we gated on CD4^+^ CD25^+^ CD69^+^, CD8^+^ CD25^+^ CD69^+^, and CD4^+^ FOLR4^+^ CD25^+^ CD69^+^ events. Oxaliplatin treatment did not cause any changes in the proportion of activated CD4^+^ T cells (a, b). However, a significant increase in the proportion of CD8^+^ CD69^+^, but not CD8^+^ CD25^+^ was observed following oxaliplatin treatment when compared to the vehicle-treated group (c, d). Oxaliplatin caused a significant increase in the proportion of CD4^+^ FOLR4^+^ CD69^+^, but not CD4^+^ FOLR4^+^ CD25^+^ Tregs, when compared to the vehicle-treated group (e, f). B cells were identified by gating on CD45^+^ TCR*β*^−^ B220^+^ cells. Oxaliplatin treatment caused a significant reduction in the proportion of B cells when compared to the vehicle-treated cohort (g). Vehicle: n=5-9; oxaliplatin: n=5-14; *∗∗P*<0.01.

To determine whether T cells were activated, we gated on CD4^+^, CD8^+^, and FOLR4^+^ expressing cells double-positive for the activation markers CD25 and CD69. There were no significant differences in the proportion of activated CD3^+^ CD4^+^ CD25^+^ T cells following oxaliplatin treatment (0.04 ± 0.02%; n=5) when compared to the vehicle-treated cohort (0.08 ± 0.02%; n=5) (Figure 3(a)). Furthermore, there were no significant differences in the proportion of activated CD3^+^ CD4^+^ CD69^+^ T cells following oxaliplatin treatment (0.42 ± 0.03%; n=5) compared to vehicle-treated control (0.4 ± 0.03%; n=5) (Figure 3(b)). Similarly, no significant differences in the proportion of activated CD3^+^ CD8^+^ CD25^+^ T cells were observed following oxaliplatin treatment (0.08 ± 0.04%; n=5) when compared to the vehicle-treated cohort (0.18 ± 0.04%; n=5) (Figure 3(c)). Conversely, a significant increase in the proportion of activated CD3^+^ CD8^+^ CD69^+^ T cells was noted following oxaliplatin treatment (0.23 ± 0.05%,* P*<0.01; n=5) when compared to the vehicle-treated cohort (0.1 ± 0.001%; n=5) (Figure 3(d)). Tregs were identified as CD4^+^/CD25^+^/FOLR4^+^ cells. CD4^+^ cells express high levels of FOLR4 as well as Foxp3 and, thus, FOLR4 can be used in substitute to Foxp3 [16–19]. No significant differences in the proportion of activated CD4^+^ FOLR4^+^ CD25^+^ T cells were observed following oxaliplatin treatment (0.18 ± 0.06%; n=5) when compared to the vehicle-treated control (0.18 ± 0.05%; n=5) (Figure 3(e)). However, a significant increase in the proportion of activated CD4^+^ FOLR4^+^ CD69^+^ T cells was observed following oxaliplatin treatment (2.69 ± 0.21%,* P*<0.01; n=5) when compared to the vehicle-treated cohort (1.7 ± 0.15; n=5) (Figure 3(f)).

### 3.3. Oxaliplatin Treatment Decreases B Cell Proportions in the Spleen

B cells were identified by gating on CD45^+^ TCR*β*^−^ B220^+^ cells. Oxaliplatin treatment caused a significant reduction in the proportion of B cells (23.1 ± 2.4%,* P*<0.0001; n=4) when compared to the vehicle-treated cohort (49 ± 0.7%; n=4) (Figure 3(g)).

### 3.4. Oxaliplatin Has No Effects on Macrophage Phenotypes or Pro-/Anti-Inflammatory Cytokines in the Spleen

**Figure 4:**
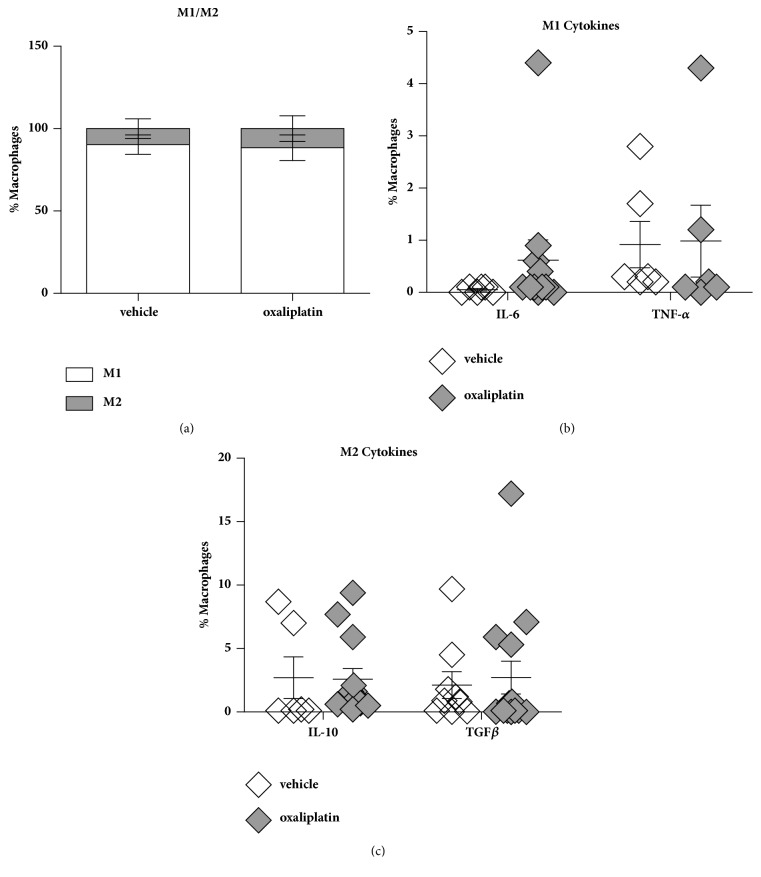
*Effects of oxaliplatin on the proportion of M1/M2 phenotypes and pro-/anti-inflammatory cytokines within the spleen*. To determine any changes in the proportions of pro-inflammatory and anti-inflammatory macrophages, a set of gating strategies were used. M1 macrophages were gated on CD45^+^ CD11B^+^, Ly6G^+^ Ly6C^+^, CD11C^+^ MHC-II^+^, CD206^+^ CD45^+^ cells. M2 macrophages were gated on: CD45^+^ CD11B^+^, Ly6G^+^ CD45^+^, CD11B^+^ MHC-II^+^, CD206^+^ CD45^+^ cells. To investigate any changes to pro-inflammatory cytokine expression cells were gated on their expression of M1 phenotypes versus IL-6, TNF-*α*, IL-10 and TGF*β*. To determine the expression of anti-inflammatory cytokines from M2 macrophages cells were gated on their phenotype versus IL-10 or TGF-*β*. No differences in M1 macrophages were observed between the vehicle-treated and oxaliplatin-treated animals (a). No differences were observed in M2 macrophages amongst the vehicle-treated and oxaliplatin-treated animals (a). No changes in M1 or M2 cytokines were observed between the vehicle-treated and oxaliplatin-treated mice (b, c). Vehicle: n=9; oxaliplatin: n=14.

To determine changes in immune cell populations within the spleen we profiled M1/M2 macrophages as well as the expression of proinflammatory (IL-6 and TNF-*α*) and anti-inflammatory (IL-10 and TGF*β*) cytokines. To determine any changes in the proportions of proinflammatory and anti-inflammatory macrophages and cytokines (pro-inflammatory: IL-6 and TNF-*α*; anti-inflammatory: IL-10 and TGF*β*), a set of gating strategies were used. M1 macrophages were gated based on CD45^+^ CD11B^+^, Ly6G^+^ Ly6C^+^, CD11C^+^ MHC-II^+^, and CD206^+^ expressing cells. M2 macrophages were gated based on: CD45^+^ CD11B^+^, Ly6G^+^ CD45^+^, CD11B^+^ MHC-II^+^, and CD206^+^ expressing cells. There were no differences in M1 macrophages amongst the vehicle-treated (90.3 ± 1.9%; n=6) and oxaliplatin-treated (88.4 ± 2.0%; n=14) groups (Figure 4(a)). Furthermore, no differences were observed in M2 macrophages amongst the vehicle-treated (9.6 ± 1.5%; n=6) and oxaliplatin-treated groups (11.5 ± 2.0%; n=14) (Figure 4(a)). Consequently, there were no changes in M1 cytokines between vehicle-treated (IL-6: 0.05 ± 0.01%; n=9; TNF-*α*: 0.91 ± 0.29%; n=9) and oxaliplatin-treated mice (IL-6: 0.61 ± 0.34%, n=14; TNF-*α*: 0.98 ± 0.45%; n=14) (Figure 4(b)). Moreover, there were no differences in M2 cytokines amongst the vehicle-treated (IL-10: 2.7 ± 1.07%; n=9; TGF*β*: 2.1%  ± 0.84%; n=9) and oxaliplatin-treated mice (IL-10: 2.5 ± 0.80%; n=14; TGF*β*: 2.7 ± 1.2%; n=14) (Figure 4(c)).

### 3.5. Effects of Oxaliplatin on Inflammation-Associated Genes in the Spleen

**Figure 5:**
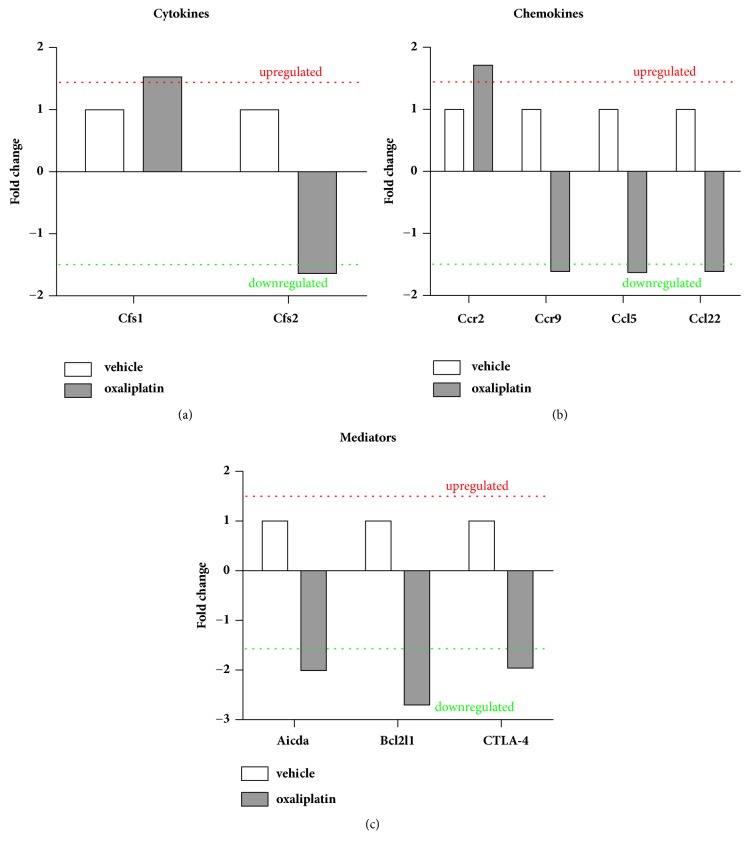
*Effects of oxaliplatin on inflammation-associated genes within the spleen*. RT^2^ profiler arrays were used to determine changes in expression of inflammation-associated genes. Oxaliplatin treatment resulted in upregulation of Csf1 and downregulation of Csf2 (a). Oxaliplatin treatment caused the upregulation of Ccr2 and downregulation of Ccr9, Ccl5 and Ccl22 gene expression (b). Oxaliplatin treatment downregulated mRNA expression levels of Aicda, Bcl2l1 and CTLA-4 (c).

To determine changes in expression of inflammation-associated genes in the spleen, RT-PCR was performed using RT^2-^ PCR-arrays. Oxaliplatin treatment caused the upregulation of the cytokine colony stimulating factor 1 (Csf-1; 1.53 fold change) but a decrease in Csf-2 (-1.64 fold change), IL-1*β* (-2.00 fold change), IL-10 (-1.62 fold change), and IL-12*β* (-2.97 fold change) (Figure 5(a)). Furthermore, oxaliplatin treatment caused the upregulation of the C-C motif chemokine receptor 2 (Ccr2) gene (1.71 fold change) but downregulated the genes Ccr5 (-1.63 fold change), C-C motif chemokine ligand Ccl5 (-1.77 fold change), Ccl22 (-1.68 fold change), and Ccr9 (-1.61 fold change) (Figure 5(b)). Furthermore, oxaliplatin treatment downregulated the genes Activation-induced cytdidine deaminase (Aicda; -2.01 fold change), Bcl-2-like 1 (Bcl2l1; -2.75 fold change), and cytotoxic T lymphocyte-associated protein 4 (CTLA-4; -1.96 fold change) (Figure 5(c)). Most genes represented on the array showed no detectable difference in expression (less than 1.5 fold) when comparing the oxaliplatin-treated group to the vehicle-treated group.

### 3.6. Oxaliplatin Treatment Increases CD8^+^ Single-Positive Thymocytes with No Effects on CD4^+^, or CD4^+^ CD8^+^ Double-Positive Populations

**Figure 6:**
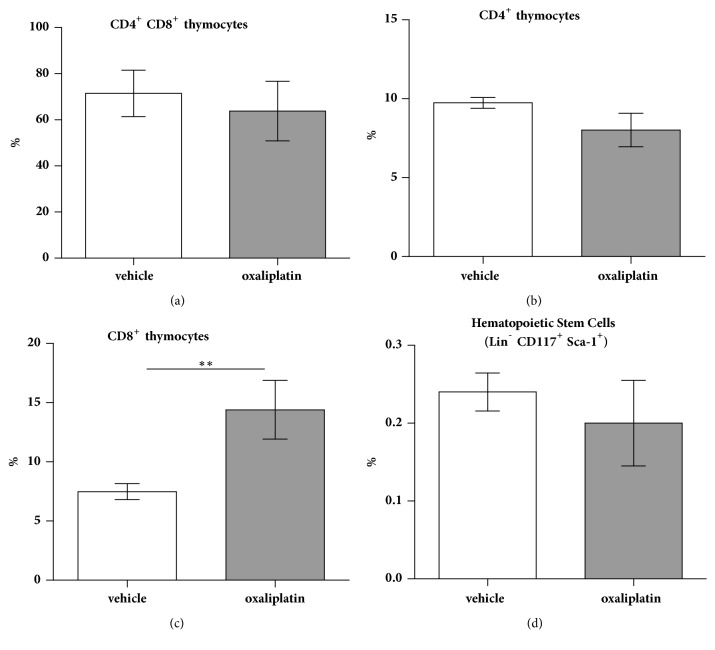
*Effects of oxaliplatin treatment on T cell populations within the thymus and progenitor cells in the bone marrow*. To determine any changes in the proportions of double-positive and single-positive thymocytes, we gated on CD4^+^ CD8^+^ events. No significant changes were observed in the proportion of double-positive CD4^+^ CD8^+^ thymocytes following oxaliplatin treatment when compared to the vehicle-treated cohort (a). No significant differences were observed in the proportion of single-positive CD4^+^ thymocytes following oxaliplatin treatment when compared to the vehicle-treated cohort (b). Oxaliplatin treatment caused a significant increase in the proportion single-positive CD8^+^ thymocytes following oxaliplatin treatment when compared to the vehicle-treated cohort (c). To determine any changes in the proportions of bone marrow hematopoietic stem and progenitor cells, we gated on Lin^−^/CD117^+^/Sca-1^+^ events. There were no significant differences in the proportion of Lin^−^/CD117^+^/Sca-1^+^ cells following oxaliplatin treatment when compared to the vehicle-treated cohort (d). N=5/group; *∗∗P*<0.01.

To assess changes in the proportions of double-positive and single-positive thymocytes, cells were gated by CD4^+^ CD8^+^ populations. No significant differences were observed in the proportion of double-positive CD4^+^ CD8^+^ thymocytes following oxaliplatin treatment (63.8 ± 5.7%; n=5) when compared to the vehicle-treated cohort (71.4 ± 4.5%; n=5) (Figure 6(a)). Furthermore, no significant differences were observed in the proportion of single-positive CD4^+^ thymocytes following oxaliplatin treatment (8.0 ± 1.06%; n=5) when compared to the vehicle-treated cohort (9.7 ± 0.3%; n=5) (Figure 6(b)). Oxaliplatin treatment caused a significant increase in the proportion single-positive CD8^+^ thymocytes following oxaliplatin treatment (14.4 ± 2.4%; n=5) when compared to the vehicle-treated cohort (7.4 ± 0.6%; n=5) (Figure 6(c)).

### 3.7. Oxaliplatin Treatment Has No Demonstrable Effects on Bone Marrow Hematopoietic Stem and Progenitor Cells

To ascertain whether changes in the proportions of bone marrow hematopoietic stem and progenitor cells, cells were gated on Lin^−^/CD117^+^/Sca-1^+^ cells. No significant differences were observed in the proportion of Lin^−^/CD117^+^/Sca-1^+^ cells following oxaliplatin treatment (0.2 ± 0.05%; n=5) when compared to the vehicle-treated cohort (0.24 ± 0.02%; n=5) (Figure 6(d)).

## 4. Discussion

This study is amongst the first to determine systemic immune responses following oxaliplatin treatment in the mouse spleen, thymus, and bone marrow. Our data show that oxaliplatin does not cause systemic immunosuppression and, in fact, can skew proinflammatory immune responses. The presentation of DAMPs following oxaliplatin treatment has previously been shown to induce immunogenic cell death in colorectal tumor cell lines [1]. Despite the potential to induce immunogenic cell death, oxaliplatin did not cause any inflammatory responses within the gastrointestinal tract [15]. Thus, it was hypothesised that this may be due to tissue-specific immune responses within the gastrointestinal tract and that immunological responses may differ systemically.

The spleen functions are to clear aged erythrocytes, filter blood-borne pathogens, antigens, and foreign materials, and play a major role in augmenting appropriate systemic immune responses [20, 21]. As the spleen receives a large volume of blood, this organ may be particularly vulnerable to platinum-based anticancer agents or, perhaps, may be a site for generating immunological responses to chemotherapy. In this study, we have shown that oxaliplatin treatment caused a significant decrease in spleen size and in the proportion of CD45^+^ immune cells. Previous work investigating spleen size following anticancer chemotherapy is conflicting. Computed tomography imaging of spleens from patients undergoing carboplatin/paclitaxel or cisplatin/etopisode chemotherapy and concomitant radiotherapy for nonsmall cell lung carcinoma demonstrate a decrease in spleen volume in 66% and 79% of patients respectively [22]. Patient spleen size is typically estimated by multiplying organ length by width and height. Furthermore, splenomegaly has been observed in colorectal cancer patients receiving oxaliplatin in a FOLFOX regimen [23–25]. Aside from our data, it is unclear how platinum-based drugs affect spleen size when given as a single agent and, thus, further work is required to understand these changes in organ size.

Despite a reduction in spleen size and cellularity following oxaliplatin treatment, the proportions of overall CD4^+^ and CD8^+^ T cells were increased in this cohort when compared to the vehicle-treated group. Helper CD4^+^ T cells play a role in adaptive immunity by conditioning the environment and, essentially, modulating the activity of other immune cells through cytokine production such as polarizing DCs that can perform cross-presentation to CD8^+^ T cells [26, 27]. There are limited studies regarding the effects of oxaliplatin and the predecessor platinum-based agents on CD4^+^ T cells. Studies investigating the effects of other anticancer agents such as cyclophosphamide have shown that this drug can selectively deplete Tregs and restore effector T cell function which is imperative for antitumor responses, as well as mounting appropriate immune responses to antigens [28, 29]. Previous work has shown that cisplatin given in combination with a TLR9 agonist CpG and a pan-human leukocyte antigen DR binding epitope enhances systemic CD4^+^ T cell responses against papillomavirus 16 E7 tumors [30]. Moreover, cisplatin treatment also leads to an increase in CD4^+^ T and CD8^+^ T cell-mediated immune responses leading to nephrotoxicity [31]. These data demonstrate the immunostimulatory potential of platinum-based drugs to mount anti-tumor responses but highlight the fact that they may also mediate tissue injury.

CD8^+^ T cells play a role in cell-mediated cytotoxicity through cytokine release, death ligand stimulation, and perforin/granzyme B-mediated pathways. In this present study, we have shown that oxaliplatin treatment increases the overall proportion of CD8^+^ T cells, and enhances CD8^+^ T cell activation as demonstrated by CD69 expression [32–34]. These data show that CD8^+^ T cells have been primed and activated as a result of appropriate antigen-presentation. Our study is in line with earlier reports which had shown that increased CD8^+^ T cell activation and function following oxaliplatin treatment in peripheral blood and colon cancer cell lines [1, 2]. Previous work assessing peripheral neuropathy following oxaliplatin treatment in C57BL/6J mice has demonstrated an increase in circulating CD8^+^ T cells [35]. Although CD8^+^ T cells were not measured in the blood, T cells primed and activated within the spleen migrate to sites of damage. Furthermore, the addition of cisplatin to an immunotherapy vaccine comprised of calreticulin and papillomavirus 16 E7 antigens for the treatment of cervical cancer enhances CD8^+^ T cell responses [36]. The activation states of CD8^+^ T cells are further supported by the downregulation of CTLA-4 gene expression observed in this study. This gene codes for the CTLA-4 inhibitory ligand which is a negative regulator of T cell function [37–39]. Thus, it is becoming well known that platinum-based agents can induce T cell responses that would be beneficial for cancer treatment; however, it is currently unknown where these CD8^+^ T cells will migrate from the spleen in response to oxaliplatin treatment and whether a subset of these cells are MAIT cells (requires tetramer which is unavailable to us) and this requires further work.

Tregs are well known for their immunosuppressive roles in maintaining self-tolerance and in controlling inflammatory responses [40, 41]. In this study, we showed that oxaliplatin treatment caused a reduction in the proportion of Tregs when compared to the vehicle-treated cohort. However, the proportion of activated Tregs following oxaliplatin treatment increased. Our findings are in contrast to a study which demonstrated an increase of Tregs in blood samples from patients receiving combined oxaliplatin and 5-fluorouracil treatment for CRC [42]. It is unclear why a decrease in Tregs is observed following oxaliplatin treatment, but the addition of 5-flourouracil to the treatment may induce differential immune responses [43–45].

Oxaliplatin has demonstrated immunostimulatory potential, and we aimed to investigate whether treatment with this drug could stimulate cytokine production in the spleen. As both cancer and oxaliplatin treatment have immunomodulatory properties, it is important to investigate their impact individually before studying them in combination. In this study, we observed the increased expression of the proinflammatory cytokine TNF-*α* in splenic T cells. Most research has demonstrated that platinum-based drugs induce TNF-*α* production by nonimmune cells. Previous studies showed that oxaliplatin treatment causes astrocyte and glial cell activation and the production of TNF-*α* in a rat model of peripheral neuropathy [46]. Additionally, an increase in TNF-*α* expression by spinal glial cells has also been observed in a model of oxaliplatin-induced neuropathic cold allodynia [47]. In addition to this, an increase in TNF-*α* has been previously described following cisplatin treatment in kidney proximal tubule and epithelial cells [48, 49]. It is known that TNF-*α* can alter neuronal function and induce cell death via the extrinsic apoptosis pathway (death receptor-mediated cascades) [50]. TNF-*α* binding to the TNF superfamily receptor recruits the caspases 8/10 to the death domain docking site and initiates the apoptotic cascade for the cleavage of caspase-3. The extrinsic and intrinsic apoptotic pathways can have some crossover and it is unclear whether death receptor stimulation has played a role in initiating the apoptotic cascade. We have found that platinum from oxaliplatin accumulates within the brain (unpublished observation). Oxaliplatin is a bulky drug that was originally thought to be too big to pass through the blood brain barrier. However, it is well known that proinflammatory cytokines can alter blood-brain barrier permeability which could allow for oxaliplatin accumulation [51–53]. Cytokine-mediated reduction in blood-brain barrier integrity could be implicated in the platinum accumulation within the brain; however, further work is required to elucidate this concept. No differences in the expression of the anti-inflammatory cytokines IL-10 and TGF*β* was observed following oxaliplatin treatment. This suggests that no anti-inflammatory responses are being initiated to counteract the increased proportion of activated CD4^+^ and CD8^+^ T cells and TNF-*α* production.

We have shown that oxaliplatin is particularly cytotoxic to splenic B cells and caused the downregulation of the Aicda gene expression, which plays a role B cell proliferation an Ig class-switching [54, 55]. Within the spleen, B cells capture antigens within the blood though complement receptors, and can initiate T cell-dependent/independent responses [56]. Research has shown that B cells can impede T cell activation by expressing IgA, IL-10, and programmed cell death ligand 1 (PD-L1) and by promoting T cell conversion to Tregs [57–61]. The overall reduction in B cells and Aicda following oxaliplatin treatment may affect IgA, IL-10, and PD-L1 production and, thus, enable optimal T cell activation. As spleen mass was significantly reduced following oxaliplatin treatment despite strong T cell responses, the depletion of B cells may account for the change in organ size. It should be noted that we have not investigated whether oxaliplatin treatment affects the connective tissue or the red pulp of the spleen, as this would also have an impact on organ size.

Macrophages within the spleen play a role in antigen recognition, processing and presentation to T cells, to ultimately mount an appropriate immune response [62, 63]. In this study, we have shown that oxaliplatin treatment has no effects on the proportion of M1/M2 macrophages, or their proinflammatory and anti-inflammatory cytokines. M1 macrophages produce the proinflammatory cytokines IL-6 and TNF-*α* upon activation, whereas the M2 phenotype expresses the anti-inflammatory cytokines IL-10 and TGF*β* [64, 65]. Splenic macrophages are in close contact with T cells and, thus, their location favours rapid antigen presentation [66, 67]. Whether changes in macrophage cytokine profiles have occurred in earlier stages remains unknown, but the increase in activated T cells suggests sufficient antigen presentation has occurred.

In addition, we show that oxaliplatin treatment differentially affects chemokine receptors and ligand expression within the spleen. Oxaliplatin treatment caused an increase in Ccr2 expression. Ccr2 is highly expressed on macrophages, and ligation by Ccl2 induces the recruitment of peripheral monocytes during infectious and inflammatory conditions [68]. As there were no changes in Ccl2 expression despite upregulated Ccr2 within the spleen following oxaliplatin treatment, this may explain why no changes were observed in the proportion of splenic macrophages. Elevated Ccr2 and Ccl2 expression have been previously observed in the dorsal root ganglia following the administration of another anticancer agent, paclitaxel, and this was positively correlated with peripheral neuropathy [69]. The effects of oxaliplatin treatment on splenic macrophages largely remain unknown and, thus, it is difficult to compare results from our current study. Furthermore, in our current study we demonstrated that oxaliplatin treatment increases Csf-1 expression within the spleen. Csf-1 plays a role in macrophage maturation, proliferation and survival [70, 71]. This cytokine is produced by a number of cells, including but not limited to, macrophages, T cells, tumor cells, epithelial cells, and endothelial cells [72, 73]. Proinflammatory cytokines such as IL-1*β*, IL-6, and TNF-*α* can also stimulate the upregulation of Csf-1 [72]. A number of studies have demonstrated the importance of Csf-1 and its effects on antigen presenting cells and T cell-mediated immunity [74–77]. Csf-1 is now given adjuvantly with the anticancer chemotherapeutics oxaliplatin, gemcitabine, levofolinate, docetaxel, and 5-fluorouracil [78, 79]. Thus, the increase in Csf-1 following oxaliplatin treatment may also potentiate T-cell mediated immunity. Furthermore, Csf-2 is a potent stimulator of granulocytes and lymphocytes [80]. However, oxaliplatin treatment appeared to downregulate its expression within the spleen. Csf-2 has the capacity to stimulate both Th1 and Th2 responses [81]. Given that its expression at the mRNA level is downregulated this may impact the production of other cytokines. IL-1*β* and IL-12*β* are proinflammatory cytokines which can potentiate T cell responses [82–84]. The gene expressions of both cytokines were reduced following oxaliplatin treatment, despite robust T cell responses observed within the spleen. Leukocytes do not contain intracellular cytokine reserves and, thus, their production is regulated transcriptionally [85]. As strong T cell responses were observed within the spleen, it is unclear whether changes at the mRNA level for IL-1*β* and IL-12*β* had occurred at an earlier stage during oxaliplatin treatment. Future studies would seek to measure IL-12*β* in purified DCs to determine the source of these changes. Similarly, reduced IL-10 mRNA expression was detected following oxaliplatin treatment, although no changes were observed in intracellular cytokine expression within the spleen from flow cytometry experiments. Similarly, IL-1*β* and IL-12*β* were also shown to be downregulated in the colon of these mice following oxaliplatin treatment [15].

Moreover, Ccr5, Ccl5, and Ccl22 play a role in lymphocyte trafficking [86, 87]. It is unclear whether the downregulation of these ligands will impact T cell migration, despite their proportional increase and activated states in the spleen. Of interest, we also noted Ccl5 and Ccl22 were downregulated in the colon [15]. Furthermore, oxaliplatin treatment reduced Ccr9 expression within the spleen. Ccr9 is typically involved in lymphocyte migration and cell survival, but it has also been implicated in antiapoptotic cascades in several pathological conditions [88–90]. The downregulation of this chemokine receptor following oxaliplatin treatment may therefore enable apoptotic signaling cascades. Oxaliplatin treatment also led to a reduction in Bcl2l1 expression, a gene involved in antiapoptotic cascades [91, 92]. The decreased expression of Ccr9 and Bcl2l1 may contribute to spleen toxicity following oxaliplatin treatment.

Our current work has shown that oxaliplatin treatment increases the proportion of single-positive CD8^+^ thymocytes, with no effect on double-positive CD4^+^ CD8^+^ thymocytes, or single-positive CD4^+^ T cells. In the thymus, lymphoid progenitors develop T cell receptor expression and become double-positive for CD4^+^ and CD8^+^ T cells [93]. Within the medullary region of the thymus, epithelial cells present MHC-I and MHC-II molecules to double-positive T cells. Thymocytes will then differentiate into single-positive CD4^+^ T cells or CD8^+^ T cells if they respond to MHC-II or MHC-I molecules, respectively [93]. Upon single-positive selection, these thymocytes migrate to secondary locations such as the spleen and lymph nodes [94, 95]. The increase in single-positive CD8^+^ thymocytes following oxaliplatin may suggest the enhanced recruitment of cytotoxic T cells to the periphery.

Furthermore, our data has shown that oxaliplatin does not negatively impact the bone marrow hematopoietic stem cell progenitor pool. There is limited research demonstrating the effects of oxaliplatin treatment on bone marrow progenitors. However, an indirect measure of bone marrow suppression caused by oxaliplatin is the onset of thrombocytopenia. Sensitivity reactions to oxaliplatin treatment have been previously associated with immune thrombocytopenia which is the Ig-mediated destruction of platelets thought to be caused by mild bone marrow suppression [96, 97]. We did not measure platelets in this study, but as the proportion of bone marrow progenitors from the oxaliplatin-treated group was similar to the vehicle-treated cohort, it does not appear oxaliplatin is immunosuppressive.

Whilst we have demonstrated that oxaliplatin-treatment can alter systemic immune responses in cancer naïve mice, our future studies will investigate the immune responses to oxaliplatin in combination with a xenograft animal model. It is well known that chemotherapeutic drugs and cancers can modulate immune responses, so it is important to elucidate their effects on the immune system exclusively, and in combination.

## 5. Conclusion

The data provide evidence that oxaliplatin can induce beneficial antitumor immune responses and that it is not an immunosuppressive agent. Our data also reveals tissue-specific immunological responses to oxaliplatin treatment.

## Figures and Tables

**Table 1:** Antibodies used for flow cytometry experiments in this study.

**Cells**	**Primary antibody**	**Conjugate**	**Host species**	**Dilution**
**Pan-leukocyte marker**	CD45	PerCP/Cy5.5	Mouse	1:400

**Pan-T cell marker**	CD3	Alexa Fluor 488	Mouse	1:400

**T cell receptor**	TCR*β*	APC	Rat	1:250

**Granulocytes**	GR-1, CD11b	PE-Cy7	Rat	1:100

**Cytotoxic T cells**	CD8	Brilliant Violet 421	Rat	1:100

**Helper T cells**	CD4	Brilliant Violet 500	Rat	1:100

**Regulatory T cells**	Folate receptor 4	Alexa Fluor 647	Mouse	1:100

**Activated T cells**	CD25	PE-Cy7	Mouse	1:100

**Activated T cells**	CD69	APC-Cy7	Mouse	1:100

**B cells**	B220	FITC	Mouse	1:400

**Macrophages**	CD11b, Ly6C, Ly6G, CD206, F4/80	PE	Rat	1:200

**Hematopoietic Stem and Progenitor Cell**	CD34	FITC	Mouse	1:100

**Hematopoietic Stem and Progenitor Cell**	c-Kit	PE	Mouse	1:100

**Hematopoietic Stem and Progenitor Cell**	Sca-1	PE-Cy7	Mouse	1:100

**Hematopoietic Stem and Progenitor Cell**	Lineage cocktail	APC	Mouse	1:100

**Cytokines**				

**IL-6**	IL-6	APC	Mouse	1:100

**TNF-** **α**	TNF-*α*	Brilliant Violet™ 510	Mouse	1:100

**IL-10**	IL-10	APC	Mouse	1:100

**TGF** **β**	TGF*β*	Brilliant Violet™ 421	Mouse	1:100

## Data Availability

The data used to support the findings of this study are available from the corresponding author upon request.

## References

[1] Tesniere A., Schlemmer F., Boige V. (2010). Immunogenic death of colon cancer cells treated with oxaliplatin. *Oncogene*.

[2] Krysko D. V., Garg A. D., Kaczmarek A., Krysko O., Agostinis P., Vandenabeele P. (2012). Immunogenic cell death and DAMPs in cancer therapy. *Nature Reviews Cancer*.

[3] Bezu L., Gomes-de-Silva L. C., Dewitte H. (2015). Combinatorial strategies for the induction of immunogenic cell death. *Frontiers in Immunology*.

[4] Yamano T., Kubo S., Fukumoto M. (2016). Whole cell vaccination using immunogenic cell death by an oncolytic adenovirus is effective against a colorectal cancer model. *Molecular Therapy - Oncolytics*.

[5] Schenk M., Mueller C. (2008). The mucosal immune system at the gastrointestinal barrier. *Best Practice & Research Clinical Gastroenterology*.

[6] Spahn T. W., Kucharzik T. (2004). Modulating the intestinal immune system: the role of lymphotoxin and GALT organs. *Gut*.

[7] Vighi G., Marcucci F., Sensi L., Di Cara G., Frati F. (2008). Allergy and the gastrointestinal system. *Clinical & Experimental Immunology*.

[8] Bronte V., Pittet M. J. (2013). The spleen in local and systemic regulation of immunity. *Immunity*.

[9] Mebius R. E., Kraal G. (2005). Structure and function of the spleen. *Nature Reviews Immunology*.

[10] Renn C. L., Carozzi V. A., Rhee P., Gallop D., Dorsey S. G., Cavaletti G. (2011). Multimodal assessment of painful peripheral neuropathy induced by chronic oxaliplatin-based chemotherapy in mice. *Molecular Pain*.

[11] Elias D., Matsuhisa T., Sideris L. (2004). Heated intra-operative intraperitoneal oxaliplatin plus irinotecan after complete resection of peritoneal carcinomatosis: Pharmacokinetics, tissue distribution and tolerance. *Annals of Oncology*.

[12] Robinson A. M., Stojanovska V., Rahman A. A., McQuade R. M., Senior P. V., Nurgali K. (2016). Effects of oxaliplatin treatment on the enteric glial cells and neurons in the mouse ileum. *Journal of Histochemistry & Cytochemistry*.

[13] McQuade R. M., Carbone S. E., Stojanovska V. (2016). Role of oxidative stress in oxaliplatin-induced enteric neuropathy and colonic dysmotility in mice. *British Journal of Pharmacology*.

[14] Moore J. P., Sakkal S., Bullen M. L. (2013). A flow cytometric method for the analysis of macrophages in the vascular wall. *Journal of Immunological Methods*.

[15] Stojanovska V., McQuade R. M., Fraser S. (2018). Oxaliplatin-induced changes in microbiota, TLR4+ cells and enhanced HMGB1 expression in the murine colon. *PLoS ONE*.

[16] Walker L. S. K. (2007). Regulatory T cells: Folate receptor 4: a new handle on regulation and memory?. *Immunology & Cell Biology*.

[17] Liang S. C., Moskalenko M., Van Roey M., Jooss K. (2013). Depletion of regulatory T cells by targeting folate receptor 4 enhances the potency of a GM-CSF-secreting tumor cell immunotherapy. *Clinical Immunology*.

[18] Yamaguchi T., Hirota K., Nagahama K. (2007). Control of immune responses by antigen-specific regulatory T cells expressing the folate receptor. *Immunity*.

[19] Kunisawa J., Hashimoto E., Ishikawa I., Kiyono H., Zimmer J. (2012). A pivotal role of vitamin B9 in the maintenance of regulatory T cells in vitro and in vivo. *PLoS ONE*.

[20] Cesta M. F. (2006). Normal structure, function, and histology of the spleen. *Toxicologic Pathology*.

[21] Bronte V., Pittet M. J. (2014). The spleen in local and systemic regulation of immunity. *Immunity*.

[22] Wen S. W., Everitt S. J., Bedő J. (2015). Spleen volume variation in patients with locally advanced non-small cell lung cancer receiving platinum-based chemo-radiotherapy. *PLoS ONE*.

[23] Angitapalli R., Litwin A. M., Kumar P. R. G. (2009). Adjuvant FOLFOX chemotherapy and splenomegaly in patients with stages II-III colorectal cancer. *Oncology*.

[24] Overman M. J., Maru D. M., Charnsangavej C. (2010). Oxaliplatin-mediated increase in spleen size as a biomarker for the development of hepatic sinusoidal injury. *Journal of Clinical Oncology*.

[25] Jung E. J., Ryu C. G., Kim G. (2012). Splenomegaly during oxaliplatin-based chemotherapy for colorectal carcinoma. *Anticancer Research*.

[26] Luckheeram R. V., Zhou Ru. I., Verma A. Dev., Xia B. (2012). CD4^+^T cells: differentiation and functions. *Clinical and Developmental Immunology*.

[27] Zhu J., Paul W. E. (2008). CD4 T cells: fates, functions, and faults. *Blood*.

[28] Awwad M., North R. J. (1988). Cyclophosphamide (Cy)-facilitated adoptive immunotherapy of a Cy-resistant tumour. Evidence that Cy permits the expression of adoptive T-cell mediated immunity by removing suppressor T cells rather than by reducing tumour burden. *The Journal of Immunology*.

[29] Weir G. M., Hrytsenko O., Stanford M. M. (2014). Metronomic cyclophosphamide enhances HPV16E7 peptide vaccine induced antigen-specific and cytotoxic T-cell mediated antitumor immune response. *OncoImmunology*.

[30] Song L., Yang M., Knoff J., Wu T., Hung C., van Hall T. (2014). Cancer immunotherapy employing an innovative strategy to enhance CD4+ T cell help in the tumor microenvironment. *PLoS ONE*.

[31] Liu M., Chien C., Burne-Taney M. (2006). A pathophysiologic role for T lymphocytes in murine acute cisplatin nephrotoxicity. *Journal of the American Society of Nephrology*.

[32] Yuzefpolskiy Y., Baumann F. M., Penny L. A. (2016). Signaling through PD-1 on CD8 T cells is critical for antigen-independent maintenance of immune memory. *The Journal of Immunology*.

[33] Wu X., Zhang H., Xing Q. (2014). PD-1^+^ CD8^+^ T cells are exhausted in tumours and functional in draining lymph nodes of colorectal cancer patients. *British Journal of Cancer*.

[34] Liu J., Zhang S., Hu Y. (2016). Targeting PD-1 and Tim-3 pathways to reverse CD8 T-cell exhaustion and enhance ex vivo T-cell responses to autologous dendritic/tumor vaccines. *Journal of Immunotherapy*.

[35] Makker P. G., Duffy S. S., Lees J. G. (2017). Characterisation of immune and neuroinflammatory changes associated with chemotherapy-induced peripheral neuropathy. *PLoS ONE*.

[36] Tseng C.-W., Hung C.-F., Alvarez R. D. (2008). Pretreatment with cisplatin enhances E7-Specific CD8+ T-cell -mediated antitumor immunity induced by DNA vaccination. *Clinical Cancer Research*.

[37] Buchbinder E. I., Desai A. (2016). CTLA-4 and PD-1 pathways: similarities, differences, and implications of their inhibition. *American Journal of Clinical Oncology*.

[38] Walker L. S. K., Sansom D. M. (2015). Confusing signals: recent progress in CTLA-4 biology. *Trends in Immunology*.

[39] Hannani D., Vétizou M., Enot D. (2015). Anticancer immunotherapy by CTLA-4 blockade: obligatory contribution of IL-2 receptors and negative prognostic impact of soluble CD25. *Cell Research*.

[40] Pellerin L., Jenks J. A., Bégin P., Bacchetta R., Nadeau K. C. (2014). Regulatory T cells and their roles in immune dysregulation and allergy. *Immunologic Research*.

[41] Lee J. H., Wang C., Kim C. H. (2009). FoxP3+ regulatory T cells restrain splenic extramedullary myelopoiesis via suppression of hemopoietic cytokine-producing T cells. *The Journal of Immunology*.

[42] Maeda K., Hazama S., Tokuno K. (2011). Impact of chemotherapy for colorectal cancer on regulatory T-cells and tumor immunity. *Anticancer Research*.

[43] Wang J. (2016). 5-Fluorouracil targets thymidylate synthase in the selective suppression of TH17 cell differentiation. *Oncotarget*.

[44] Kobayashi R., Yoshimatsu K., Yokomizo H., Katsube T., Ogawa K. (2007). Low-dose chemotherapy with leucovorin plus 5-fluorouracil for colorectal cancer can maintain host immunity. *Anticancer Reseach*.

[45] Ghiringhelli F., Bruchard M., Apetoh L. (2013). Immune effects of 5-fluorouracil: Ambivalence matters. *OncoImmunology*.

[46] Jung Y., Lee J. H., Kim W., Yoon S. H., Kim S. K. (2017). Anti-allodynic effect of Buja in a rat model of oxaliplatin-induced peripheral neuropathy via spinal astrocytes and pro-inflammatory cytokines suppression. *BMC Complementary and Alternative Medicine*.

[47] Kim C., Lee J. H., Kim W. (2016). The suppressive effects of Cinnamomi Cortex and its phytocompound coumarin on oxaliplatin-induced neuropathic cold allodynia in rats. *Molecules*.

[48] Ramesh G., Brian Reeves W. (2009). Cisplatin increases TNF-*α* mRNA stability in kidney proximal tubule cells. *Renal Failure*.

[49] Ramesh G., Kimball S. R., Jefferson L. S., Reeves W. B. (2007). Endotoxin and cisplatin synergistically stimulate TNF-*α* production by renal epithelial cells. *American Journal of Physiology-Renal Physiology*.

[50] Fulda S., Debatin K. M. (2006). Extrinsic versus intrinsic apoptosis pathways in anticancer chemotherapy. *Oncogene*.

[51] Pan W., Stone K. P., Hsuchou H., Manda V. K., Zhang Y., Kastin A. J. (2011). Cytokine signaling modulates blood-brain barrier function. *Current Pharmaceutical Design*.

[52] Rochfort K. D., Collins L. E., Murphy R. P., Cummins P. M. (2014). Downregulation of blood-brain barrier phenotype by proinflammatory cytokines involves NADPH oxidase-dependent ROS generation: consequences for interendothelial adherens and tight junctions. *PLoS ONE*.

[53] Troletti C. D., de Goede P., Kamermans A., de Vries H. E. (2016). Molecular alterations of the blood–brain barrier under inflammatory conditions: the role of endothelial to mesenchymal transition. *Biochimica et Biophysica Acta (BBA) - Molecular Basis of Disease*.

[54] Park S.-R. (2012). Activation-induced cytidine deaminase in B cell immunity and cancers. *Immune Network*.

[55] Heltemes-Harris L. M., Gearhart P. J., Ghosh P., Longo D. L. (2008). Activation-induced deaminase-mediated class switch recombination is blocked by anti-IgM signaling in a phosphatidylinositol 3-kinase-dependent fashion. *Molecular Immunology*.

[56] Pillai S., Cariappa A. (2009). The follicular versus marginal zone B lymphocyte cell fate decision. *Nature Reviews Immunology*.

[57] Liu Y. (2015). B cells can suppress chemotherapy-induced immunogenic cell death. *Cancer Discovery*.

[58] Shalapour S., Font-Burgada J., Di Caro G. (2015). Immunosuppressive plasma cells impede T-cell-dependent immunogenic chemotherapy. *Nature*.

[59] Schwartz M., Zhang Y., Rosenblatt J. D. (2016). B cell regulation of the anti-tumor response and role in carcinogenesis. *Journal for ImmunoTherapy of Cancer*.

[60] Tadmor T., Zhang Y., Cho H.-M., Podack E. R., Rosenblatt J. D. (2011). The absence of B lymphocytes reduces the number and function of T-regulatory cells and enhances the anti-tumor response in a murine tumor model. *Cancer Immunology, Immunotherapy*.

[61] Olkhanud P. B., Damdinsuren B., Bodogai M. (2011). Tumor-evoked regulatory B cells promote breast cancer metastasis by converting resting CD4^+^ T cells to T-regulatory cells. *Cancer Research*.

[62] Asano K., Nabeyama A., Miyake Y. (2011). CD169-positive macrophages dominate antitumor immunity by crosspresenting dead cell-associated antigens. *Immunity*.

[63] Miyake Y., Asano K., Kaise H., Uemura M., Nakayama M., Tanaka M. (2007). Critical role of macrophages in the marginal zone in the suppression of immune responses to apoptotic cell-associated antigens. *The Journal of Clinical Investigation*.

[64] Martinez F. O., Gordon S. (2014). The M1 and M2 paradigm of macrophage activation: time for reassessment. *F1000Prime Reports*.

[65] De Palma M., Lewis C. E. (2013). Macrophage regulation of tumor responses to anticancer therapies. *Cancer Cell*.

[66] Borges da Silva H., Fonseca R., Pereira R. M., Cassado A. d., Álvarez J. M., D’Império Lima M. R. (2015). Splenic macrophage subsets and their function during blood-borne infections. *Frontiers in Immunology*.

[67] Backer R., Schwandt T., Greuter M. (2010). Effective collaboration between marginal metallophilic macrophages and CD8+ dendritic cells in the generation of cytotoxic T cells. *Proceedings of the National Acadamy of Sciences of the United States of America*.

[68] Shi C., Pamer E. G. (2011). Monocyte recruitment during infection and inflammation. *Nature Reviews Immunology*.

[69] Zhang H., Boyette-Davis J. A., Kosturakis A. K. (2013). Induction of monocyte chemoattractant protein-1 (mcp-1) and its receptor ccr2 in primary sensory neurons contributes to paclitaxel-induced peripheral neuropathy. *The Journal of Pain*.

[70] Otero K., Turnbull I. R., Poliani P. L. (2009). Macrophage colony-stimulating factor induces the proliferation and survival of macrophages via a pathway involving DAP12 and *β*-catenin. *Nature Immunology*.

[71] Burgess A. W., Metcalf D. (1980). The nature and action of granulocyte - macrophage colony stimulating factors. *Blood*.

[72] Griffin J. D., Cannistra S. A., Demetri G. D., Ernst T. J., Kanakura Y., Sullivan R. (1990). The biology of GM-CSF: Regulation of production and interaction with its receptor. *The International Journal of Cell Cloning*.

[73] Zisman E., Waisman A., Ben-Yair E., Tartakovsky B. (1993). Production of colony-stimulating factor 1 by T cells: Possible involvement in their interaction with antigen-presenting cells. *Cytokine*.

[74] Wada H., Noguchi Y., Marino M. W., Dunn A. R., Old L. J. (1997). T cell functions in granulocyte/macrophage colony-stimulating factor deficient mice. *Proceedings of the National Acadamy of Sciences of the United States of America*.

[75] Mach N., Dranoff G. (2000). Cytokine-secreting tumor cell vaccines. *Current Opinion in Immunology*.

[76] Dranoff G., Jaffee E., Lazenby A. (1993). Vaccination with irradiated tumor cells engineered to secrete murine granulocyte-macrophage colony-stimulating factor stimulates potent, specific, and long-lasting anti-tumor immunity. *Proceedings of the National Acadamy of Sciences of the United States of America*.

[77] Gillessen S., Naumov Y. N., Nieuwenhuis E. E. (2003). CD1d-restricted T cells regulate dendritic cell function and antitumor immunity in a granulocyte-macrophage colony-stimulating factor-dependent fashion. *Proceedings of the National Acadamy of Sciences of the United States of America*.

[78] Correale P., Botta C., Rotundo M. S. (2014). Gemcitabine, oxaliplatin, levofolinate, 5-fluorouracil, granulocyte-macrophage colony-stimulating factor, and interleukin-2 (GOLFIG) versus FOLFOX chemotherapy in metastatic colorectal cancer patients: The GOLFIG-2 multicentric open-label randomized phase III trial. *Journal of Immunotherapy*.

[79] Locke F., Clark J. I., Gajewski T. F. (2010). A phase II study of oxaliplatin, docetaxel, and GM-CSF in patients with previously treated advanced melanoma. *Cancer Chemotherapy and Pharmacology*.

[80] Khatami S., Brummer E., Stevens D. A. (2001). Effects of granulocyte-macrophage colony stimulating factor (GM-CSF) in vivo on cytokine production and proliferation by spleen cells. *Clinical & Experimental Immunology*.

[81] Shi Y., Liu C. H., Roberts A. I. (2006). Granulocyte-macrophage colony-stimulating factor (GM-CSF) and T-cell responses: what we do and don't know. *Cell Research*.

[82] Wesa A. K., Galy A. (2001). IL-1 beta induces dendritic cells to produce IL-12. *International Immunology*.

[83] Ben-Sasson S. Z., Hu-Li J., Quiel J. (2009). IL-1 acts directly on CD4 T cells to enhance their antigen-driven expansion and differentiation. *Proceedings of the National Acadamy of Sciences of the United States of America*.

[84] Lyakh L., Trinchieri G., Provezza L., Carra G., Gerosa F. (2008). Regulation of interleukin-12/interleukin-23 production and the T-helper 17 response in humans. *Immunological Reviews*.

[85] James S. P. (2001). Detection of cytokine mRNA expression by PCR. *Current Protocols in Immunology*.

[86] Pinho V., Oliveira S. H., Souza D. G. (2003). The role of CCL22 (MDC) for the recruitment of eosinophils during allergic pleurisy in mice. *Journal of Leukocyte Biology*.

[87] Borish L. C., Steinke J. W. (2003). Cytokines and chemokines. *The Journal of Allergy and Clinical Immunology*.

[88] Sharma P. (2014). CCR9-mediated inhibition of drug-induced apoptosis in prostate cancer cells. *Cancer Research*.

[89] Huang Y., Wang D., Wang X. (2016). Abrogation of CC chemokine receptor 9 ameliorates ventricular remodeling in mice after myocardial infarction. *Scientific Reports*.

[90] Sharma P. K., Singh R., Novakovic K. R., Eaton J. W., Grizzle W. E., Singh S. (2010). CCR9 mediates PI3K/AKT-dependent antiapoptotic signals in prostate cancer cells and inhibition of CCR9-CCL25 interaction enhances the cytotoxic effects of etoposide. *International Journal of Cancer*.

[91] Janumyan Y. M., Sansam C. G., Chattopadhyay A. (2003). Bcl-xL/Bcl-2 coordinately regulates apoptosis, cell cycle arrest and cell cycle entry. *EMBO Journal*.

[92] Hagenbuchner J., Ausserlechner M. J., Porto V. (2010). The anti-apoptotic protein BCL2L1/Bcl-xL is neutralized by pro-apoptotic PMAIP1/noxa in neuroblastoma, thereby determining bortezomib sensitivity independent of prosurvival MCL1 expression. *The Journal of Biological Chemistry*.

[93] Germain R. N. (2002). T-cell development and the CD4-CD8 lineage decision. *Nature Reviews Immunology*.

[94] Vanhecke D., Leclercq G., Plum J., Vandekerckhove B. (1995). Characterization of distinct stages during the differentiation of human CD69+CD3+ thymocytes and identification of thymic emigrants. *The Journal of Immunology*.

[95] Xu X., Ge Q. (2014). Maturation and migration of murine cd4 single positive thymocytes and thymic emigrants. *Computational and Structural Biotechnology Journal*.

[96] Curtis B. R., Kaliszewski J., Marques M. B. (2006). Immune-mediated thrombocytopenia resulting from sensitivity to oxaliplatin. *American Journal of Hematology*.

[97] Woo H. S., Lee K. H., Yoon P. H. (2015). Oxaliplatin-induced immune-mediated thrombocytopenia: A case report. *Cancer Research and Treatment*.

